# Myeloid-CITED2 Deficiency Exacerbates Diet-Induced Obesity and Pro-Inflammatory Macrophage Response

**DOI:** 10.3390/cells12172136

**Published:** 2023-08-24

**Authors:** Atif Zafar, Hang Pong Ng, E. Ricky Chan, Sally L. Dunwoodie, Ganapati H. Mahabeleshwar

**Affiliations:** 1 Department of Pathology, Case Western Reserve University School of Medicine, Cleveland, OH 44106, USA; 2 Cleveland Institute for Computational Biology, Case Western Reserve University School of Medicine, Cleveland, OH 44106, USA; 3 Victor Chang Cardiac Research Institute, Sydney, NSW 2010, Australia; 4 School of Clinical Medicine, Faculty of Medicine and Health, UNSW, Sydney, NSW 2052, Australia

**Keywords:** signal transduction, gene regulation, macrophages, inflammation, pathogenesis

## Abstract

Macrophages are the principal component of the innate immune system that are found in all tissues and play an essential role in development, homeostasis, tissue repair, and immunity. Clinical and experimental studies have shown that transcriptionally dynamic pro-inflammatory macrophages are involved in the pathogenesis of diet-induced obesity and insulin resistance. However, cell-intrinsic mechanisms must exist that bridle uncontrolled pro-inflammatory macrophage activation in metabolic organs and disease pathogenesis. In this study, we show that CBP/p300-interacting transactivator with glutamic acid/aspartic acid-rich carboxyl-terminal domain 2 (CITED2) is an essential negative regulator of pro-inflammatory macrophage activation and inflammatory disease pathogenesis. Our in vivo studies show that myeloid-CITED2 deficiency significantly elevates high-fat diet (HFD)-induced expansion of adipose tissue volume, obesity, glucose intolerance, and insulin resistance. Moreover, myeloid-CITED2 deficiency also substantially augments HFD-induced adipose tissue inflammation and adverse remodeling of adipocytes. Our integrated transcriptomics and gene set enrichment analyses show that CITED2 deficiency curtails BCL6 signaling and broadly elevates BCL6-repressive gene target expression in macrophages. Using complementary gain- and loss-of-function studies, we found that CITED2 deficiency attenuates, and CITED2 overexpression elevates, inducible BCL6 expression in macrophages. At the molecular level, our analyses show that CITED2 promotes BCL6 expression by restraining STAT5 activation in macrophages. Interestingly, siRNA-mediated knockdown of STAT5 fully reversed elevated pro-inflammatory gene target expression in CITED2-deficient macrophages. Overall, our findings highlight that CITED2 restrains inflammation by promoting BCL6 expression in macrophages, and limits diet-induced obesity and insulin resistance.

## 1. Introduction

Macrophages are major phagocytic cells of the innate immune system that play a critical role in maintaining tissue homeostasis and protecting the host from the deleterious effects of infections and injuries [1]. Macrophages sense the surrounding environment by an array of cell surface receptors, including pattern recognition receptors, scavenger receptors, Siglecs (sialic-acid-binding immunoglobulin-like lectins), and integrins. Activation of these cell surface receptors spurs complex intracellular signaling pathways that engage several transcription factors, such as NFkB, STATs, IRFs, and KLFs, to shape the macrophage inflammatory response to extremal stimuli [2]. However, unrestrained macrophage activation leads to the development of inflammatory diseases such as asthma, arthritis, atherosclerosis, vasculitis, psoriasis, colitis, and ileitis [3]. Interestingly, clinical and experimental studies provide evidence that inflammatory macrophage accumulation in white adipose tissue plays a critical role in the development of diet-induced obesity and type 2 diabetes mellitus (T2D) [4]. These classically activated macrophages are found in higher numbers in adipose tissues of obese subjects than in lean subjects [5]. They are the major sources of pro-inflammatory cytokines such as TNF, IL1β, and IL6 [6]. These macrophage-derived pro-inflammatory cytokines can function in a paracrine or endocrine manner to cause decreased insulin sensitivity in major metabolic organs [7]. Despite the extensive studies in this area highlighting the implicit role of inflammatory macrophages in the development of obesity and insulin resistance, the cell-intrinsic negative regulatory mechanisms that restrain the inflammatory response in macrophages remain elusive. Thus, identifying such regulatory mechanisms that bridle pro-inflammatory macrophage activation could aid the development of effective therapeutics for the treatment of co-morbidities of obesity, such as insulin resistance and T2D.

The pro-inflammatory gene expression at the transcriptional level is restrained by several inducible negative regulators such as B cell lymphoma 6 (BCL6) and IκBα to bridle the inflammatory response [8]. The proto-oncogene BCL6 encodes a transcriptional repressor protein containing an N-terminal POZ domain and a C-terminal zinc finger domain [9]. BCL6 has been shown to play a crucial role in maintaining macrophage quiescence and limiting systemic inflammatory response [10]. Indeed, several studies have shown that BCL6 restrains pro-inflammatory gene expression by cooperating with other co-repressor factors such as nuclear receptor co-repressor 1 (NCOR1), NCOR2, histone deacetylases (HDACs), BCL6 corepressor (BCOR), and SIN3A [11]. Interestingly, BCL6 inhibits TLR-mediated pro-inflammatory cytokine and chemokine expression in macrophages through proximal binding to NFκB response elements [10]. Further, recent studies have uncovered that BCL6 negatively regulates atherogenic gene expression in vivo and loss of BCL6 in bone marrow accelerates inflammation and/or cholesterol-dependent atherosclerosis [12]. In addition, high-fat diet (HFD)-fed mice with BCL6-knockout bone marrow transplantation also exhibited significantly elevated inflammatory gene expression in white adipose tissue [12]. Interestingly, BCL6 overexpression significantly attenuated HFD-induced body weight gain, hepatic lipid deposition, inflammatory macrophage accumulation, and pro-inflammatory gene expression in liver tissue [13]. More importantly, BCL6 overexpression substantially curtailed HFD-induced insulin resistance and lowered the serum glucose profile [13]. Despite previous studies demonstrating the importance of BCL6 in regulating inflammatory gene expression, the precise role of BCL6 signaling in metabolic inflammation and diet-induced metabolic dysfunction is not well understood. In this study, we found that myeloid Cbp/p300-interacting transactivator with Glu/Asp-rich carboxy-terminal domain 2 (CITED2) promotes BCL6 signaling to limit the expression of inflammatory gene targets and the pathogenesis of diet-induced obesity and metabolic dysfunction.

CITED2 is a member of the CBP/p300-interacting transactivator with Glu/Asp-rich carboxy-terminal domain family of transcriptional regulators and is mainly localized in the nucleus [14]. Our previous studies have shown that CITED2 plays an important role in cellular development and differentiation [15]. Experimental murine studies have shown that CITED2 mutation or deficiency results in congenital heart and neural crest defects, impairment in the development of the liver, lung, adrenal tissue, placenta, and perturbation in the left–right patterning of the body axis [16]. Our prior studies showed that CITED2 is primarily expressed in murine and human macrophages [17]. Myeloid-CITED2-specific knockdown mice are highly prone to lipopolysaccharide (LPS)-induced endotoxic shock syndrome, zymosan-induced lung inflammation, and experimental atherogenesis [17,18,19]. Mechanistically, our prior studies showed that CITED2 in cooperation with PPARγ promotes anti-inflammatory gene expression while suppressing NFκB, HIF1α, STAT1, and IRF1 signaling to attenuate pro-inflammatory gene expression in macrophages [17,18,19]. Moreover, a previous report has shown that CITED2 regulates GCN5-mediated PGC-1a acetylation and hepatic gluconeogenesis in murine studies [20]. Despite previous studies indicating a role for hepatic-CITED2 in gluconeogenesis, the importance of myeloid-CITED2 signaling in diet-induced obesity and insulin resistance has not been investigated. Herein, we provide evidence that myeloid-CITED2 limits pro-inflammatory gene expression by elevating BCL6 signaling, and substantially curtails diet-induced obesity and insulin resistance.

## 2. Materials and Methods

### 2.1. Reagents and Resources

Key reagents and resources, including the source of antibodies, chemicals, reagents, and primer sequence information, are provided in Tables S1 and S2 in the Supplementary Materials.

### 2.2. Experimental Mouse Models

All animal procedures were approved by the Case Western Reserve University Institutional Animal Care and Use Committee and conformed to the American Association for Accreditation of Laboratory Animal Care guidelines. The investigators performing the animal experiments were blinded to mouse genotypes by noncontinuous ear tag numbering. The mice colony was maintained in pathogen-free conditions. The mouse room was on a 12 h light/dark cycle and standard laboratory chow diet. Myeloid-CITED2-deficient mice were generated in our laboratory as described in our previous study [17,21]. In brief, the control (*Lyz2^cre^*) and myeloid-specific CITED2-deficient (*Cited2^fl/fl^:Lyz2^cre^*) mice were generated by breeding male and female *Cited2^fl/fl^* and *Lyz2^cre^* mice. The Cited2fl/fl:Lyz2cre mice (pure C57BL/6 background) contained two Cited2 floxed alleles and one Lyz2 Cre allele. Mice with one Cre allele (*Lyz2^cre^*) were used as the control group (pure C57BL/6 background). *Lyz2^cre^* and *Cited2^fl/fl^*:*Lyz2^cre^* mice were fed a HFD (D12108C, Research Diets, New Brunswick, NJ, USA) for the indicated periods to induce obesity and insulin resistance. The animal colony was maintained in pathogen-free conditions and the mouse room was on a 12 h light/dark cycle and monitored daily. An oral glucose tolerance test (GTT) was performed with 1 g/kg body weight glucose solution following an overnight unfed period, and an insulin tolerance test (ITT) was performed on mice that were unfed for 5 h by injecting insulin at 1 U/kg body weight. Blood glucose levels for GTT and ITT were monitored at indicated time points using Accu-Chek glucose readers (Roche, Basel, Switzerland). After 20 weeks of HFD, *Lyz2^cre^* and *Cited2^fl/fl^*:*Lyz2^cre^* mice were evaluated for body fat distribution using a 7T small animal MRI scanner.

Further, perigonadal visceral adipose tissues were obtained from the experimental mice after 20 weeks of control or HFD feeding. The adipose tissues were fixed in 10% buffered formalin, embedded in paraffin, and stained with hematoxylin and eosin. These hematoxylin-and-eosin-stained adipose tissue images were utilized to analyze the number and size of adipocytes using the ImageJ program (National Institutes of Health, Bethesda, MD, USA). To quantify the macrophage abundance in adipose tissue, paraffin sections of adipose tissues were deparaffinized in xylene and rehydrated in graded ethanol series. Samples were subjected to antigen retrieval steps with antigen unmasking solution. Samples were treated with 0.3% H_2_O_2_ for 30 min at room temperature and the nonspecific antibody binding was blocked by using a blocking buffer. These tissue sections were incubated with rabbit anti-F4/80 antibody overnight at 4 °C and were subsequently incubated with biotin-conjugated goat anti-rabbit IgG for 30 min at room temperature. These sections were further incubated in avidin–biotin complex reagent, and the immunostaining was visualized by using 3,3′diaminobenzidine reagent. Images were acquired using a microscope and then macrophage areas were quantified using the ImageJ program.

### 2.3. Cell Culture

The RAW264.7 cells were cultured in Dulbecco’s modified Eagle’s medium (DMEM) supplemented with 10% fetal bovine serum (FBS), 100 U/mL penicillin, 10 µg/mL streptomycin, and 2 mM glutamine in a humidified incubator (5% CO_2_ and 37 °C). The bone-marrow-derived macrophages (BMDMs) were generated from indicated mice genotypes by ex vivo differentiation of bone marrow cells. Briefly, bone marrow cells from 8-week-old *Lyz2^cre^* and *Cited2^fl/fl^*:*Lyz2^cre^* mice femurs and tibiae were collected. These bone marrow cells were cultured in complete DMEM supplemented with recombinant mouse macrophage colony-stimulating factor (M-CSF) for 7 days. The resulting BMDMs were harvested and utilized for the stated experiments. Mouse thioglycolate-elicited peritoneal macrophages (PMs) were obtained by inducing peritonitis with 3% thioglycolate broth in 8- to 12-week-old mice. These mice were euthanized on day 3, and the primary peritoneal macrophages obtained were cultured in complete DMEM as described above. The primary macrophages from adipose tissue were obtained 20 weeks after the initiation of control or HFD. Briefly, adipose tissue was washed with ice-cold 1X phosphate-buffered saline to remove red blood cells (RBCs). The adipose tissue was minced with scalpel blades and incubated in a collagenase–dispase mixture (3 mg/mL) for 4 h at room temperature with gentle shaking. The cellular suspension was collected using a tissue strainer, and anti-F4/80 microbeads were used to purify the macrophages from these cellular suspensions. The total RNA samples derived from adipose tissue macrophages (ATMs) were utilized for RT-qPCR analyses.

### 2.4. RNAseq Analysis

Total RNA from *Lyz2^cre^* and *Cited2^fl/fl^*:*Lyz2^cre^* mice BMDMs were obtained by using the High Pure RNA Isolation Kit. The RNA samples were quantified by using a Qubit Fluorometer and an Agilent 2100 BioAnalyzer was utilized to determine the quality, using a cutoff of RIN > 7.0 to select specimens for further analysis. A cDNA library for RNAseq was generated from 150 ng of total RNA using an Illumina TruSeq Stranded Total RNA kit with Ribo Zero Gold for rRNA removal according to the manufacturer’s protocol. The resulting purified mRNA was used as input for the Illumina TruSeq kit, in which libraries are tagged with unique adapter indexes. Final libraries were validated on the Agilent 2100 BioAnalyzer, quantified via qPCR, and pooled at equimolar ratios. Pooled libraries were diluted, denatured, and loaded onto an Illumina NextSeq 550 System using a high-throughput flow cell. STAR Aligner was used for mapping the sequencing reads to the mm10 mouse reference genome. The aligned reads were then analyzed with Cuffdiff to obtain gene-level expression data using the GENCODE gene annotation for mm10, and reported as fragments per kilobase per million reads mapped (FPKM). Differential expression analysis was also performed using the Cuffdiff package and significantly differentially expressed genes were defined using an adjusted *p*-value < 0.05 (FDR-corrected). Gene expression tables for relevant pairwise comparisons were analyzed for gene set enrichment analysis (GSEA) using GenePattern (Broad Institute) [22]. We specifically utilized Hallmark pathways data sets for current studies. The gene interactome and interactive gene network between significantly dysregulated HALLMARK signaling pathways were analyzed by Circos [23] and Cytoscape 3.9.1. [24], respectively. The gene sets with a family-wise error rate (FWER) value less than 0.05 were considered enriched. Heatmaps were generated using ClustVis. The sequencing data reported in this manuscript have been deposited in the Gene Expression Omnibus (GSE218217).

### 2.5. RNA Extraction, Real-Time Quantitative PCR, and Western Blot

Total RNA extracts were isolated from indicated specimens using the High Pure RNA Isolation kit. The total RNA samples (1 µg each) were reverse-transcribed using M-MuLV reverse transcriptase in the presence of random hexamers and oligo-dT primers. Real-time quantitative PCR was performed using the Universal SYBR Green PCR Master Mix or TaqMan Universal Master Mix on an Applied Biosystems Step One Plus real-time PCR system (Thermo Fisher Scientific Inc, Waltham, MA) in the presence of gene-specific primers. The list of primers utilized in this study is provided in Supplementary Materials Table S2. Macrophages were lysed in 1X RIPA buffer supplemented with protease and phosphatase inhibitors. Protein concentration in cell lysates was measured by a bicinchoninic acid (BCA) protein assay and equal amounts of protein lysates were loaded on 8% Mini-PROTEAN TGXTM precast gels (Bio-Rad). The resolved protein was transferred to nitrocellulose membranes and blotted using standard protocols with antibodies against BCL6 (1:1000); STAT3, p-STAT3, (1:2500); STAT5, p-STAT5 (1:2500); and β-ACTIN (1:5000).

### 2.6. Transient Transfection and Luciferase Reporter Assay

RAW264.7 cells and mice BMDMs were transfected using Lipofectamine transfection reagents according to the manufacturer’s instructions (Thermo Fisher Scientific Inc, Waltham, MA, USA). Transfected cells were stimulated with LPS (100 ng/mL) or PBS and used for indicated experiments. RAW264.7 cells were transfected with native BCL6 promoter-driven luciferase reporter plasmid (pLA/B9 WT) or were co-transfected with CITED2-specific siRNA or pCMV-CITED2 plasmid using the Lipofectamine transfection reagent. After transfection, these cells were challenged with PBS or 100 ng/mL LPS for 18 h. Luciferase reporter activity was measured and normalized according to the manufacturer’s instructions. The results are presented as relative luciferase activity over the control group.

### 2.7. Quantitative and Statistical Analysis

All data, unless indicated otherwise, are presented as the mean ± standard deviation (SD). The statistical significance of differences between the two groups was analyzed by Student’s t-test or two-way ANOVA with Bonferroni multiple comparison tests. *p* < 0.05 was considered statistically significant.

## 3. Results

### 3.1. Myeloid-CITED2 Deficiency Exacerbates HFD-Induced Obesity and Insulin Resistance

**Figure 1:**
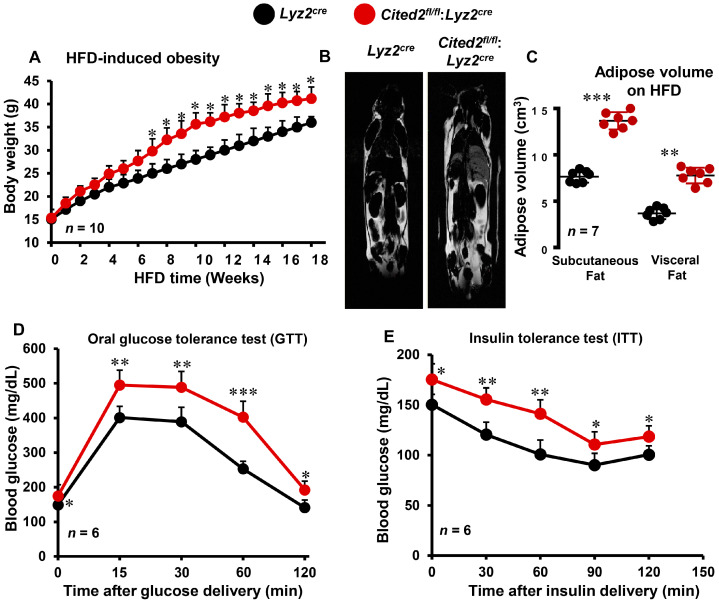
**Myeloid-CITED2 deficiency exacerbates HFD-induced obesity and insulin resistance.** (**A**) *Lyz2^cre^* and *Cited2^fl/fl^*:*Lyz2^cre^* mice were fed a HFD, and body weight gains were recorded (*n* = 10). (**B**,**C**) *Lyz2^cre^* and *Cited2^fl/fl^*:*Lyz2^cre^* mice that were fed a HFD for 20 weeks were subjected to MRI (**B**) to assess the distribution and quantification of adipose tissue volume (**C**) (*n* = 7). (**D**,**E**) *Lyz2^cre^* and *Cited2^fl/fl^*:*Lyz2^cre^* mice were subjected to an oral glucose tolerance test (**D**) or insulin tolerance test (**E**) after 18 weeks of HFD (*n* = 6). Data were analyzed using two-way ANOVA (**A**,**C**–**E**). All values are reported as means ± sd. * *p* < 0.05, ** *p* < 0.01, *** *p* < 0.001.

Prior reports have shown that macrophage-mediated adipose tissue inflammation exacerbates HFD-induced obesity and insulin resistance [25,26,27,28]. However, whether myeloid-CITED2 plays any significant role in the pathogenesis of diet-induced obesity and insulin resistance has not been examined. Our previous studies indicated that CITED2 restrains pro-inflammatory macrophage response and pathogenesis of inflammatory diseases [17,18,19]. Thus, we hypothesized that macrophage-CITED2 deficiency will augment HFD-induced obesity and insulin resistance in vivo. To test this hypothesis, cohorts of *Lyz2^cre^* and *Cited2^fl/fl^*:*Lyz2^cre^* mice were fed a HFD, and changes in body weight gains were recorded. As shown in Figure 1A, HFD feeding rapidly increased the body weight gain in *Lyz2^cre^* mice. Interestingly, *Cited2^fl/fl^*:*Lyz2^cre^* mice were highly susceptible to HFD-induced body weight gain, and maintained significantly higher body weight compared to *Lyz2^cre^* mice fed a HFD (Figure 1A). Concordant with these findings, MRI analyses showed that *Cited2^fl/fl^*:*Lyz2^cre^* mice fed a HFD exhibit significantly higher subcutaneous and visceral adipose tissue volume compared to *Lyz2^cre^* mice fed a HFD (Figure 1B,C). To explore the impact of myeloid-CITED2 deficiency on HFD-induced insulin resistance, we performed glucose and insulin tolerance tests (GTT and ITT) by utilizing *Lyz2^cre^* and *Cited2^fl/fl^*:*Lyz2^cre^* mice. Oral GTT results revealed that myeloid-CITED2-deficient mice were significantly more glucose intolerant and maintained significantly higher blood glucose levels after 18 weeks of HFD challenge (Figure 1D). This result was secondary to increased insulin resistance, as evidenced by the significantly curtailed response to the glucose-lowering effects of exogenous insulin observed in HFD-fed *Cited2^fl/fl^*:*Lyz2^cre^* mice (Figure 1E). Overall, these findings show that myeloid-CITED2 knockdown significantly exacerbates HFD-induced obesity and insulin resistance. 

### 3.2. Myeloid-CITED2 Deficiency Enhances HFD-Induced Adipose Tissue Macrophage Abundance

**Figure 2:**
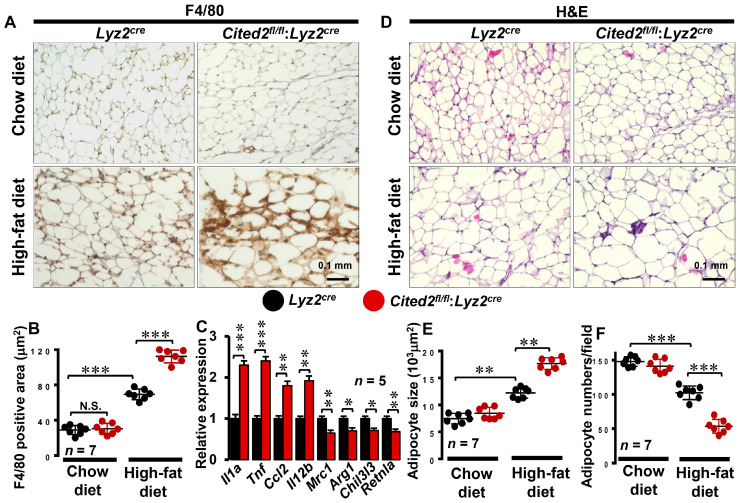
**Myeloid-CITED2 deficiency augments adipose tissue macrophage abundance.** (**A**,**B**) Perigonadal adipose tissue sections of *Lyz2^cre^* and *Cited2^fl/fl^*:*Lyz2^cre^* mice fed a chow or HFD for 18 weeks were stained for macrophages by anti-F4/80 antibody (**A**). Area (**B**) of F4/80-positive cells was quantified using ImageJ software 1.53e (*n* = 7). (**C**) Total RNA samples from HFD-fed *Lyz2^cre^* and *Cited2^fl/fl^*:*Lyz2^cre^* mouse adipose tissue macrophages were analyzed for expression of *Il1a*, *Tnf*, *Ccl2*, *Il12*, *Mrc1*, *Arg1*, *Chil3l3*, and *Retnla* by RT-qPCR (*n* = 5). (**D**–**F**) Perigonadal adipose tissue sections of *Lyz2^cre^* and *Cited2^fl/fl^:Lyz2^cre^* mice fed a chow or HFD for 18 weeks were stained for hematoxylin and eosin (**D**) to quantify adipocyte size (**E**) and number (**F**) (*n* = 7). Data were analyzed by two-way ANOVA. All values are reported as means ± sd. Scale bars, 0.1 mm. N.S. not significant; * *p* < 0.05, ** *p* < 0.01, *** *p* < 0.001.

Previous studies have shown that diet-induced obesity triggers macrophage accumulation in visceral adipose tissue and establishes chronic low-grade inflammation [29]. This chronic inflammation of adipose tissue is associated with increased insulin resistance, and impeding macrophage-specific pro-inflammatory signaling pathways improved insulin sensitivity in HFD-fed hosts [4,7]. In this context, our findings show that myeloid-CITED2 deficiency exacerbates HFD-induced obesity, subcutaneous and visceral fat accumulation, glucose intolerance, and insulin resistance in vivo (Figure 1A–E). Thus, we sought to examine whether myeloid-specific CITED2 deficiency alters HFD-induced adipose tissue macrophage abundance and adipocyte morphology in vivo. Accordingly, chow- or HFD-fed *Lyz2^cre^* and *Cited2^fl/fl^*:*Lyz2^cre^* mice visceral adipose tissues were subjected to detailed immunohistochemical and morphometric analyses. Our analyses show that myeloid-CITED2 knockdown did not alter macrophage abundance in visceral adipose tissue on the chow diet (Figure 2A,B). However, the HFD challenge significantly elevated macrophage abundance in *Lyz2^cre^* mice visceral adipose tissue (Figure 2A,B). Surprisingly, visceral adipose tissue macrophage abundance was dramatically increased in *Cited2^fl/fl^*:*Lyz2^cre^* mice fed a HFD compared to *Lyz2^cre^* mice fed a HFD (Figure 2A,B). Further, we examined whether myeloid-CITED2 deficiency alters pro- or anti-inflammatory gene expression in adipose tissue macrophages following HFD. As shown in Figure 2C, myeloid-CITED2 deficiency significantly elevated pro-inflammatory gene expression (*Il1a, Tnf, Ccl2,* and *Il12b*) while limiting anti-inflammatory (*Mrc1, Arg1, Chil3l3*, and *Retnla*) gene expression in adipose tissue macrophages. Next, we assessed whether myeloid-CITED2 deficiency alters visceral adipocyte morphometrics on a chow or HFD. Our findings show that adipocyte size and numbers were comparatively similar between *Lyz2^cre^* and *Cited2^fl/fl^*:*Lyz2^cre^* mice fed a chow diet (Figure 2D–F). As expected, HFD feeding significantly enlarged adipocyte size and attenuated adipocyte numbers in a given area (Figure 2D–F). Interestingly, *Cited2^fl/fl^*:*Lyz2^cre^* mice fed a HFD exhibited a substantial and significant increase in adipocyte size and a decrease in adipocyte numbers per field view as compared to HFD-fed *Lyz2^cre^* mice (Figure 2D–F). Collectively, our analyses show that myeloid-CITED2-deficient mice are susceptible to HFD-induced adipose tissue inflammation and adverse morphometric changes in adipocytes.

### 3.3. CITED2 Deficiency Augments Broad Pro-Inflammatory Gene Expression in Macrophages

**Figure 3:**
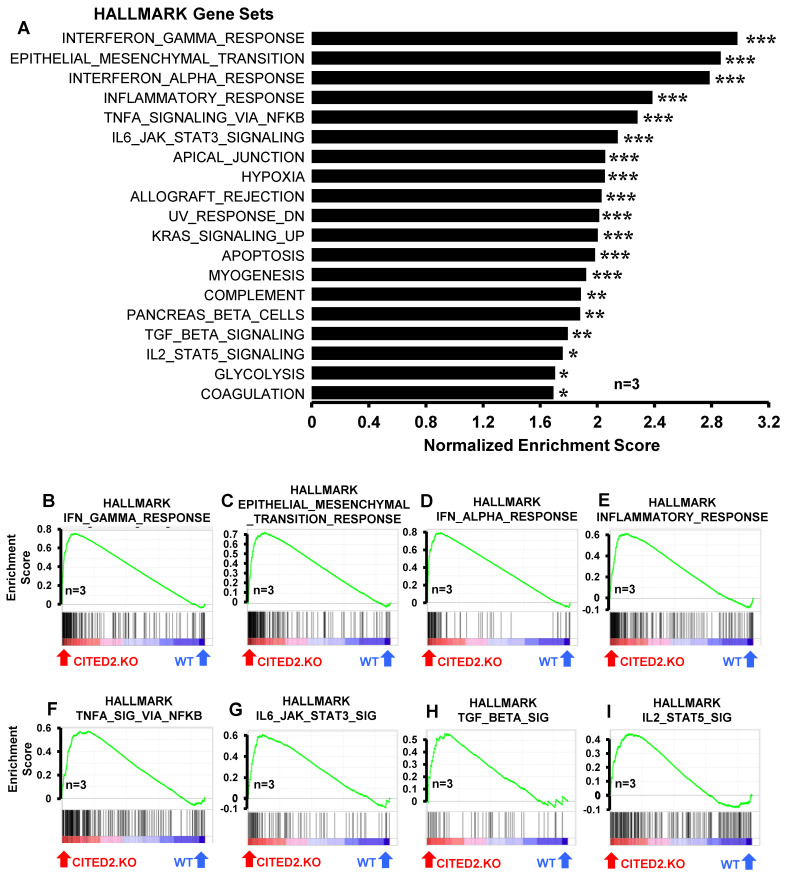
**CITED2 deficiency augments broad pro-inflammatory gene expression in macrophages.** (**A**) GSEA of RNAseq data that are altered in *Lyz2^cre^* and *Cited2^fl/fl^*:*Lyz2^cre^* mice bone-marrow-derived macrophages (BMDMs). FWER *p*-value less than 0.05 was considered significant (*n* = 3). (**B**–**I**) Enrichment plots of indicated gene set obtained by GSEA comparing *Lyz2^cre^* and *Cited2^fl/fl^*:*Lyz2^cre^* mice BMDM RNAseq data (*n* = 3). FWER *p*-value: * *p* < 0.05; ** *p* < 0.01; *** *p* < 0.001.

**Figure 4:**
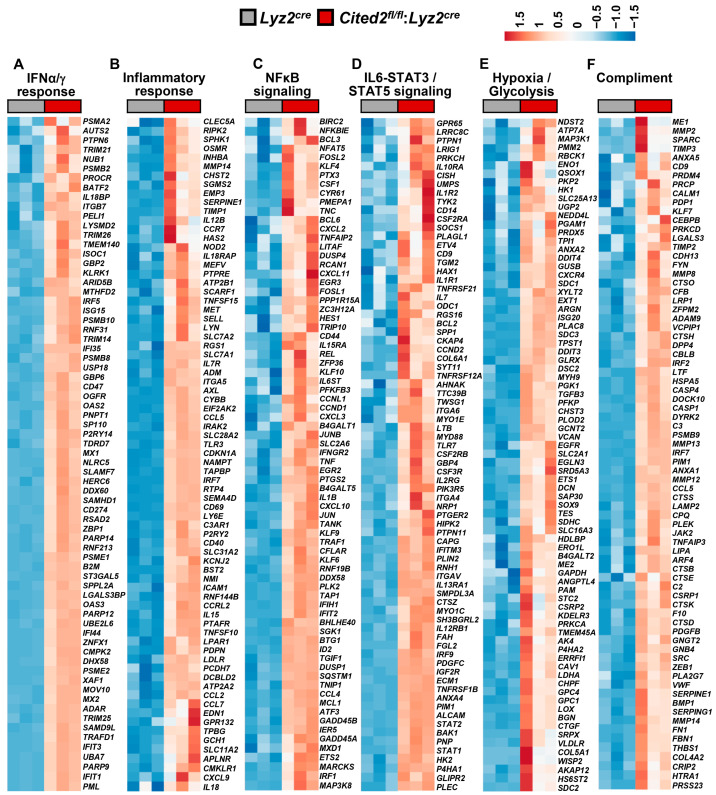
**CITED2 deficiency exacerbates pro-inflammatory gene expression in macrophages.** (**A**–**F**) Total RNA samples from *Lyz2^cre^* and *Cited2^fl/fl^*:*Lyz2^cre^* mice BMDMs were subjected to RNAseq analysis. The RNAseq data were evaluated by GSEA studies. CITED2 deficiency significantly elevated pro-inflammatory gene targets involved in IFNα/g response (**A**), inflammatory response (**B**), NFκB signaling (**C**), IL6-STAT3/STAT5 signaling (**D**), hypoxia and glycolysis response (**E**), and compliment response (**F**) in macrophages (*n* = 3). GSEA results from IFNα and IFNγ response data sets were combined to generate panel A. Similarly, IL6-STAT3 and STAT5, and hypoxia and glycolysis response data sets were combined to generate panels D and E, respectively.

Our previous studies have shown that CITED2 curbs inducible pro-inflammatory gene expression in macrophages [17,18,19]. Particularly, CITED2 limits TLR ligand- or IFNγ-induced HIF1α, NFkB, STAT1, and IRF1 signaling, and corresponding pro-inflammatory target gene expression in macrophages [17,18,19]. However, our prior analyses showed that unstimulated human/murine primary macrophages and cell lines express the CITED2 protein [17]. Thus, we hypothesized that CITED2 is an endogenous transcriptional repressor of pro-inflammatory genes in the resting state and CITED2 deficiency would be sufficient to elevate broad pro-inflammatory gene expression in unstimulated macrophages. To test this hypothesis, total RNA samples from *Lyz2^cre^* and *Cited2^fl/fl^*:*Lyz2^cre^* mice BMDMs were utilized to perform unbiased gene expression profiling analysis. The data derived from RNAseq studies were subjected to gene set enrichment analysis (GSEA) to identify CITED2-regulated pro-inflammatory signaling pathways and gene targets. Surprisingly, our analyses show that CITED2 deficiency significantly augments several pro-inflammatory pathways, including IFNγ response, epithelial–mesenchymal transition, IFNα response, inflammatory response, TNFα signaling via NFκB, IL6-JAK-STAT3 signaling, apical junction, hypoxia response, allograft rejection, UV response, KRAS signaling, apoptosis, myogenesis, complement response, TGF-β signaling, IL-2-STAT5 signaling, glycolysis, and coagulation pathways in macrophages (Figure 3A–I). Next, we cataloged the expression of pro-inflammatory genes in our RNAseq analysis. Our analysis shows that numerous IFNα and IFNγ response gene targets (*Psma2, Auts2, Ptpn6, Trim21, Nub1, Procr*, etc.) were significantly elevated in CITED2-deficient macrophages (Figure 4A). Similarly, a substantial number of inflammatory response (*Clec5a, Ripk2, Sphk1, Osmr, Inhba, Mmp14*, etc.) and NFkB signaling (*Birc2, Nfkbie, Bcl3, Nfat5, Fosl2, Klf4*, etc.) gene targets were significantly heightened in CITED2-deficient BMDMs (Figure 4B,C). Further, CITED2 deficiency also aggravated many IL6-STAT3/STAT5 signaling responses (*Gpr65, Lrrc8c, Ptpn1, Lrig1, Prkch, Il10ra*, etc.), hypoxia response/glycolysis (*Ndst2, Atp7a, Map3k1, Pmm2, Rbck1, Eno1*, etc.) and compliment response (*Me1, Mmp2, Sparc, Timp3, Anxa5, Cd9*, etc.) gene targets in macrophages (Figure 4D–F). Taken together, our findings show that CITED2 deficiency robustly augmented numerous pro-inflammatory signaling pathways and target gene expression in resting macrophages. Thus, CITED2 serves as an endogenous tonic repressor that maintains macrophage quiescence by repressing pro-inflammatory gene expression.

### 3.4. CITED2 Deficiency Derepresses Classical BCL6 Gene Targets

**Figure 5:**
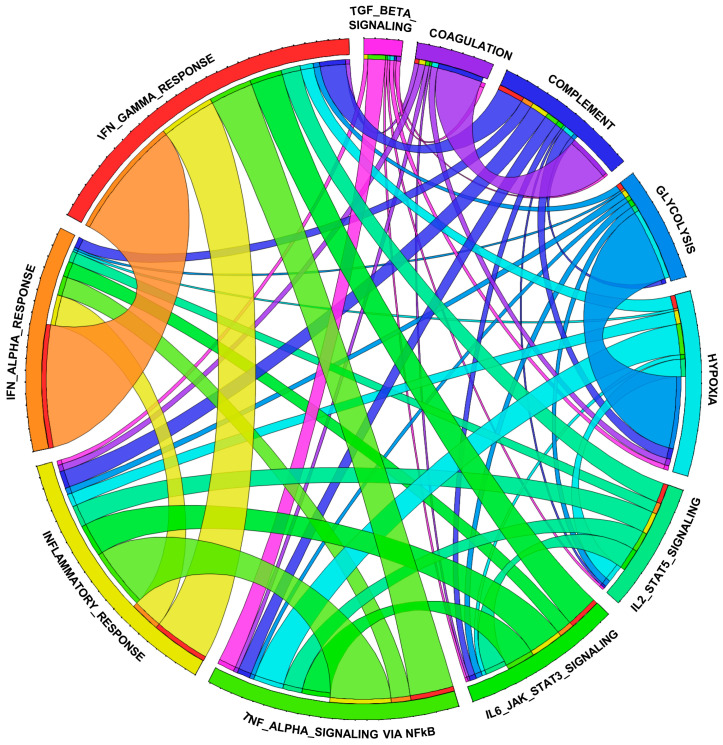
**Gene interaction between dysregulated signaling pathways in CITED2-deficient macrophages.** Gene interactome analyses between the major inflammatory signaling pathways that are upregulated in CITED2-deficient macrophages were performed by utilizing Circos. The gene interaction between inflammatory response, IFN-α response, IFN-γ response, TGF-β signaling, TNF-α signaling via NFκB, IL6-JAK-STAT3 signaling, IL2-STAT5 signaling, coagulation, complement, glycolysis, and hypoxia pathways are shown. The width of the connecting lines between pathways represents the degree of gene interactions.

**Figure 6:**
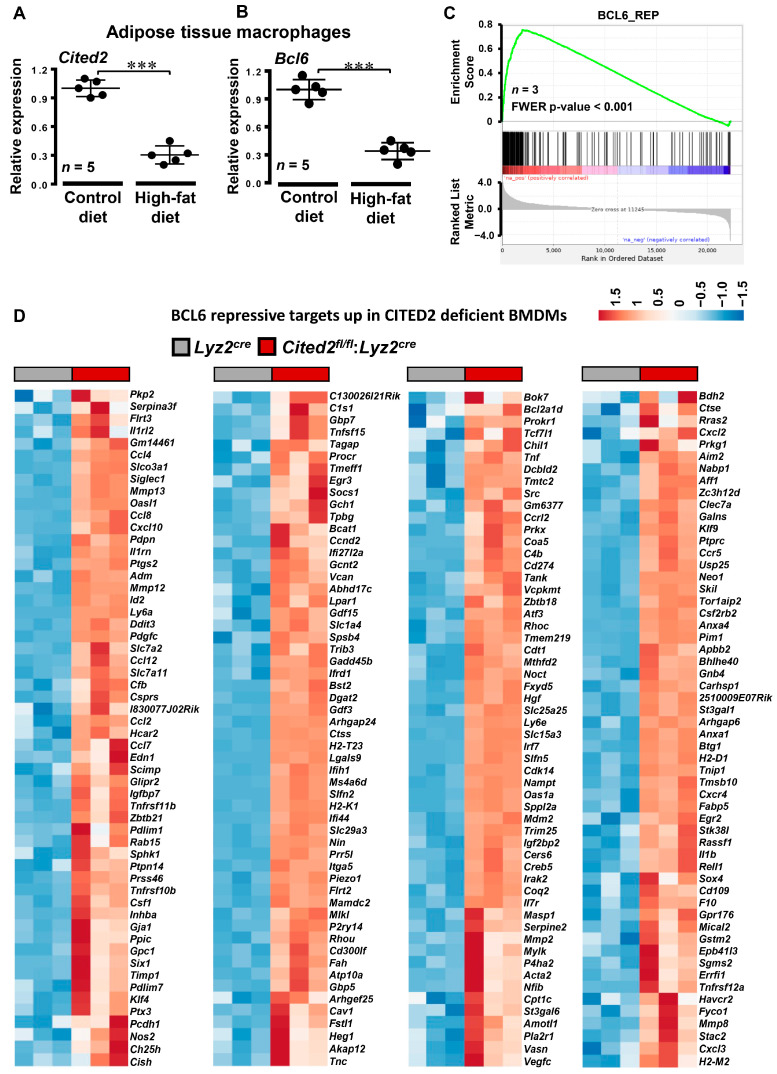
**CITED2 deficiency derepresses BCL6 gene targets in macrophages.** (**A**,**B**) Adipose tissue macrophages were obtained from *wild-type* mice fed a chow or HFD for 20 weeks. Total RNA extracts from these adipose tissue macrophages were analyzed for the expression of *CITED2* (**A**) and *Bcl6* (**B**) by RT-qPCR analyses (*n* = 5). Data were analyzed using ANOVA and all values are reported as means ± sd. *** *p* < 0.001. (**C**) Total RNA samples from *Lyz2^cre^* and *Cited2^fl/fl^*:*Lyz2^cre^* mice BMDMs were subjected to RNAseq analysis. The RNAseq data were subjected to GSEA by using BCL6 repressive gene targets. The enrichment plots of BCL6 repressive gene sets (**C**) obtained by comparing *Lyz2^cre^* and *Cited2^fl/fl^*:*Lyz2^cre^* mice BMDMs RNAseq data are shown. FWER *p*-value less than 0.05 was considered significant (*n* = 3). (**D**) Heatmaps of BCL6 repressive gene targets that are significantly upregulated in *Cited2^fl/fl^*:*Lyz2^cre^* mice BMDMs as compared to *Lyz2^cre^* mice BMDMs (*n* = 3).

**Figure 7:**
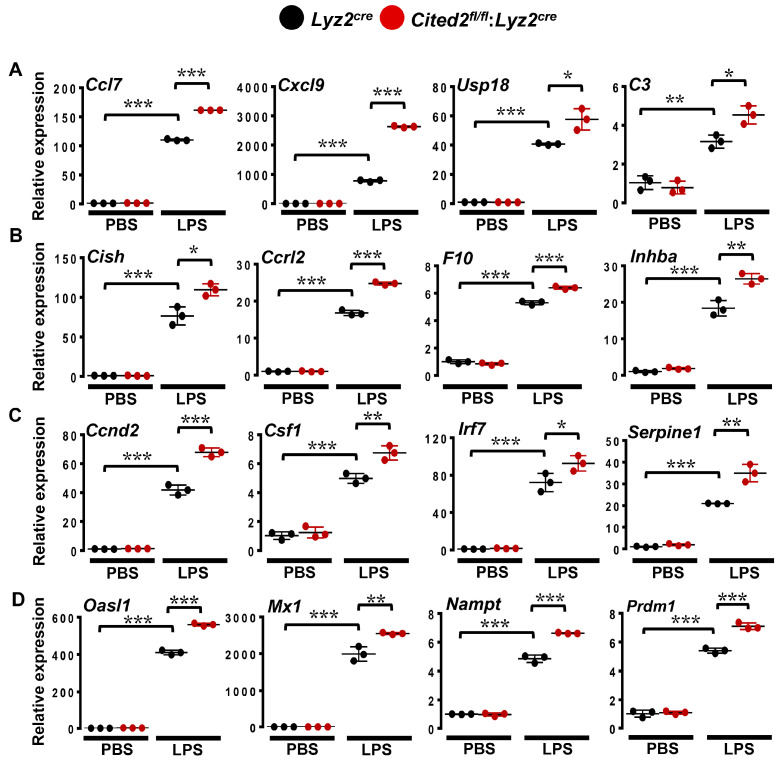
**CITED2 deficiency elevates LPS-induced BCL6 repressive gene targets in macrophages.** (**A**–**D**) *Lyz2^cre^* and *Cited2^fl/fl^*:*Lyz2^cre^* mice BMDMs were stimulated with 100 ng/mL of LPS for 4 h. Total RNA samples were evaluated in Figure 7. *Cxcl9*, *Usp18*, *C3*, (**B**) *Cish, Ccrl2*, *F10*, *Inhba*, (**C**) *Ccnd2*, *Csf1*, *Irf7*, *Serpine1*, and (**D**) *Oasl1*, *Mx1*, *Nampt*, and *Prdm1* by RT-qPCR. 36B4 was used as a housekeeping gene. All the experiments were performed in three independent triplicates. Values are reported as mean ± SD. Data were analyzed by ANOVA followed by Bonferroni post-testing. * *p* < 0.05, ** *p* < 0.01, and *** *p* < 0.001.

GSEA of RNAseq studies shows that CITED2 deficiency significantly and substantially increased several pro-inflammatory signaling pathways and gene targets in macrophages (Figure 3 and Figure 4). Given this expansive amplification of pro-inflammatory signaling pathways in CITED2-deficient macrophages, we intended to evaluate the degree of gene interaction between these signaling pathways. Our gene interactome analyses by utilizing Circos [23] showed extensive interaction between dysregulated pro-inflammatory signaling pathways (Figure 5). Further, the interaction of inflammatory pathways and gene network analyses using Cytoscape [24] also substantiated these observations (Supplementary Figure S1). Akin to these observations, previous studies have shown that BCL6 deficiency also broadly heightens pro-inflammatory signaling pathways and gene expression in macrophages [10]. Thus, we hypothesized that CITED2 may utilize BCL6 signaling to repress pro-inflammatory signaling pathways and target gene expression in macrophages. To test this notion, first, we examined the CITED2 and BCL6 expression patterns in lean and obese mice adipose tissue macrophages. Accordingly, adipose tissue macrophages were obtained from wild-type mice fed a chow or HFD for 20 weeks, and total RNA samples were analyzed for CITED2 and *Bcl6* expression. Our analyses show that HFD feeding significantly attenuated CITED2 and *Bcl6* expression in adipose tissue macrophages (Figure 6A,B). Next, we examined whether BCL6 repressive gene targets [10] were altered in CITED2-deficient macrophages by utilizing the BROAD-GSEA program. Our analyses show that BCL6 repressive gene targets (pro-inflammatory) were significantly and positively enriched in CITED2-deficient macrophages (Figure 6C). As anticipated, numerous BCL6 repressive gene targets were significantly elevated in CITED2-deficient macrophages (Figure 6D and Figure S2A–D). As shown in Figure 6D, classical pro-inflammatory BCL6 repressive gene targets, such as *Ccl4, Ccl7, Ccl8, Ccl12, Cfb, Nos2, Mmp12, Mmp2, Ptgs2,* and *Tnf,* were significantly heightened in CITED2-deficient macrophages. Further, GSEAs of BCL6 repressive gene targets that are elevated in CITED2-deficient macrophages show enrichment of classical proinflammatory pathways (Supplementary Figure S3) as observed in CITED2 null macrophages (Figure 3A). Given the importance of BCL6 signaling in restraining innate immune cell response in the pathogenesis of inflammatory disease conditions [12], we sought to determine whether CITED2 deficiency alters inducible pro-inflammatory gene target expression in macrophages. Accordingly, *Lyz2^cre^* and *Cited2^fl/fl^*:*Lyz2^cre^* mice PMs were stimulated with LPS, and expression of BCL6 repressive gene targets was evaluated by RT-qPCR analyses. We have utilized the significantly enriched and elevated BCL6 repressive targets (Figure 6C,D) as a biomarker to identify heightened pro-inflammatory gene expression response in CITED2-deficient macrophages. Our analyses show that LPS challenge significantly elevated classical pro-inflammatory gene targets, including *Ccl7, Cxcl9, Usp18, C3, Cish, Ccrl2, F10, Inhba, Ccnd2, Csf1, Irf7, Serpine1, Oasl1, Mx1, Nampt*, and *Prdm1*, in *Lyz2^cre^* mice PMs (Figure 7A–D). Remarkably, expression of these classical BCL6 repressive target genes was significantly and substantially increased in CITED2-deficient PMs challenged with LPS compared to *Lyz2^cre^* mice PMs (Figure 7A–D). Collectively, our analyses show that CITED2 deficiency attenuates BCL6 signaling and results in a broad increase in BCL6 repressive target gene expression in CITED2-deficient macrophages. 

### 3.5. CITED2 Promotes BCL6 Expression in Macrophages 

**Figure 8:**
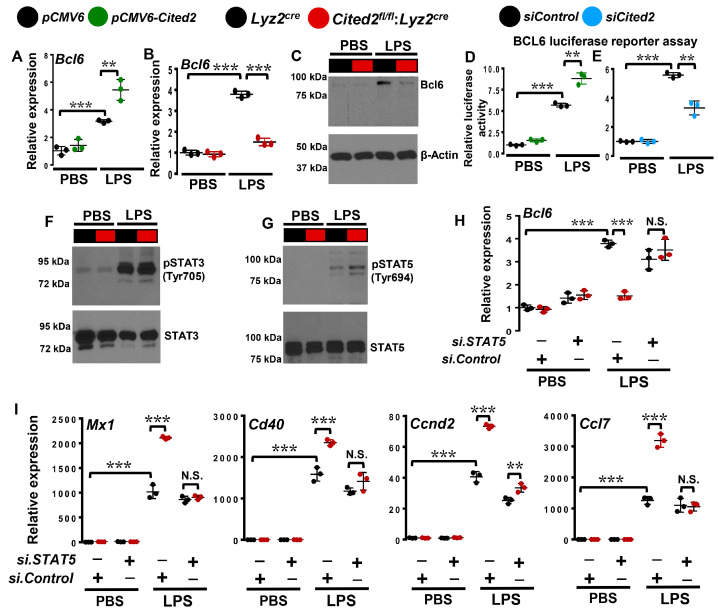
**CITED2 promotes BCL6 expression in macrophages.** (**A**–**C**) RAW264.7 cells transfected with *pCMV6* or *pCMV6-Cited2* plasmids as well as BMDMs from *Lyz2^cre^* and *Cited2^fl/fl^*:*Lyz2^cre^* mice were stimulated with 100 ng/mL of LPS for 4 h. Total RNA samples were evaluated for *Bcl6* expression by RT-qPCR (**A**,**B**) and BCL6 protein expression was analyzed by Western blot (**C**). (**D**,**E**) RAW264.7 cells were transfected with *Bcl6* promoter-driven luciferase reporter plasmid in the presence of *pCMV6-Cited2* plasmid (**D**) or *siCited2* siRNA (**E**). These cells were stimulated with 100 ng/mL of LPS for 18 h, and cell lysates were analyzed for luciferase reporter activity (*n* = 3). (**F**,**G**) *Lyz2^cre^* and *Cited2^fl/fl^*:*Lyz2^cre^* mice BMDMs were stimulated with 100 ng/mL of LPS for 4 h. Total protein extracts were analyzed for the phosphorylation and expression of STAT3 (**F**) and STAT5 (**G**) by Western blot (*n* = 3). (**H**,**I**) *Lyz2^cre^* and *Cited2^fl/fl^*:*Lyz2^cre^* mice BMDMs were transfected with control or *Stat5*-specific siRNA. These cells were stimulated with 100 ng/mL of LPS for 4 h. Total RNA samples from these experiments were analyzed for expression of *Bcl6* (**H**), *Mx1, Cd40, Ccnd2*, and *Ccl7* (**I**) by RT-qPCR (*n* = 3). Actin and 36B4 were used as housekeeping genes for Western blot and RT-qPCR analyses, respectively. Values are reported as mean ± SD. Data were analyzed by ANOVA followed by Bonferroni post-testing. N.S., not significant; ** *p* < 0.01; *** *p* < 0.001.

Our transcriptomics analyses show that several BCL6 repressive targets are positively enriched and elevated in CITED2-deficient macrophages (Figure 6C,D). Thus, we examined whether altering CITED2 levels impacts BCL6 mRNA or protein expression in macrophages. Accordingly, RAW264.7 cells overexpressing CITED2 (pCMV6-Cited2) or empty vector (pCMV6) were stimulated with LPS, and *Bcl6* expression was evaluated by quantitative PCR analysis (Figure 8A). Our analysis shows that CITED2 overexpression significantly and robustly elevated LPS-induced *Bcl6* expression in the RAW264.7 macrophage cell line (Figure 8A). Next, we examined whether CITED2 deficiency affects BCL6 expression in macrophages. To test this notion, *Lyz2^cre^* and *Cited2^fl/fl^*:*Lyz2^cre^* mice BMDMs were stimulated with LPS, and BCL6 mRNA and protein expression were determined by RT-qPCR and Western blotting, respectively. As shown in Figure 8B,C, LPS challenge significantly induced BCL6 mRNA as well as protein expression in *Lyz2^cre^* mice BMDMs. However, LPS-induced BCL6 mRNA as well as protein expression are significantly curtailed in CITED2-deficient macrophages as compared to *Lyz2^cre^* mice BMDMs (Figure 8B,C). Concurrently, we tested whether altering CITED2 modulates *Bcl6* expression at the transcriptional level. Accordingly, RAW264.7 cells were cotransfected with *Bcl6* promoter-driven luciferase reporter plasmid in the presence of pCMV6-*Cited2* or *siCited2*. These cells were challenged with LPS, and luciferase reporter activity was recorded. Our analyses show that CITED2 overexpression significantly elevated LPS-induced *Bcl6* luciferase reporter activity (Figure 8D). Concordantly, CITED2 deficiency substantially curtailed LPS-induced *Bcl6* promoter-driven luciferase reporter activity in macrophages (Figure 8E). Collectively, our study results show that CITED2 transcriptionally promotes BCL6 expression in macrophages.

### 3.6. CITED2 Deficiency Boosts STAT5-Mediated BCL6 Repression in Macrophages 

Previous studies have shown that a complex interplay between STAT3 and STAT5 signaling regulates BCL6 expression [30]. Interestingly, STAT3 activation elevates and STAT5 activation represses BCL6 expression [31]. In this context, the GSEA of our RNAseq studies shows that CITED2 deficiency alters STAT3 and STAT5 signaling in macrophages (Figure 3A,G,I). Thus, we hypothesized that dysregulated activation of STAT3 or STAT5 in CITED2-deficient macrophages may attenuate BCL6 expression. To test this notion, control as well as myeloid-CITED2-deficient BMDMs were challenged with LPS, and expression and phosphorylation status of STAT3 and STAT5 were evaluated. Our analyses show that CITED2 deficiency did not alter LPS-induced STAT3 phosphorylation (Tyr705) in macrophages (Figure 8F). However, LPS-induced STAT5 phosphorylation (Tyr 694) was significantly elevated in CITED2-deficient macrophages (Figure 8G). Therefore, we examined whether inhibiting STAT5 signaling could reverse attenuated Bcl6 expression in CITED2-deficient macrophages. Accordingly, *Lyz2^cre^* and *Cited2^fl/fl^*:*Lyz2^cre^* mice BMDMs were transfected with *Stat5*-specific siRNA. These cells were challenged with LPS, and *Bcl6* expression was analyzed by RT-qPCR (Figure 8H). As anticipated, LPS-induced *Bcl6* expression was significantly attenuated in CITED2-deficient macrophages (Figure 8H). Interestingly, Stat5 silencing was sufficient to reverse attenuated *Bcl6* expression in CITED2-deficient macrophages (Figure 8H). Next, we examined whether rescue of Bcl6 expression through *Stat5* silencing could repress elevated pro-inflammatory target gene expression in CITED2-deficient macrophages. As shown in Figure 8I, the LPS challenge significantly elevated the expression of classical pro-inflammatory gene targets such as *Mx1, Cd40, Ccnd2,* and *Ccl7* in CITED2-deficient macrophages. More importantly, Stat5 silencing significantly attenuated LPS-induced BCL6 repressive gene targets *(Mx1, Cd40, Ccnd2*, and *Ccl7)* expression in CITED2-deficient macrophages (Figure 8I). Collectively, our analyses show that targeting STAT5 signaling in CITED2-deficient macrophages rescues attenuated BCL6 levels and restrains attendant pro-inflammatory gene expression.

## 4. Discussion

Our studies show that myeloid-CITED2 restrains diet-induced obesity, insulin resistance, and adipose tissue inflammation, and curtails broad pro-inflammatory gene expression by elevating BCL6 expression in macrophages. The main findings of our study are as follows: (i) myeloid-CITED2 deficiency exacerbates HFD-induced obesity and insulin resistance; (ii) myeloid-CITED2 deficiency elevates adipose tissue inflammation and triggers adverse morphometric changes in adipocytes; (iii) CITED2 deficiency augments broad pro-inflammatory gene expression in macrophages; (iv) CITED2 deficiency derepresses classical BCL6 gene targets in macrophages; (v) CITED2 deficiency attenuates inducible BCL6 mRNA and protein expression in macrophages; (vi) CITED2 deficiency elevates STAT5 activation in macrophages; and (vii) STAT5 inhibition rescues attenuated BCL6 levels and restrains attendant pro-inflammatory gene expression in CITED2-deficient macrophages. Collectively, our analyses uncover the pleiotropic nature of CITED2 which attenuates inflammation by promoting BCL6 expression in macrophages, and substantially curbs diet-induced obesity and insulin resistance in vivo.

Macrophages are the sentinel cells of the innate immune system and play an essential role in the onset of inflammatory diseases [32]. The pathogenic responses of macrophages are predominately regulated by cellular transcriptional machinery to elicit a robust pro-inflammatory response [33]. This involves the coordinated regulation of upstream regulators, multiple transcriptional factors, and cofactors. Further, several molecular and transcriptional mechanisms exist to constrain the inflammatory response in macrophages and limit the harmful effects of inflammation. However, the molecular and transcriptional mechanisms involving negative regulation of inflammatory response are not well examined. In this context, BCL6 has been shown as a key transcriptional repressor that limits pro-inflammatory gene expression in macrophages [10]. Specifically, multiple studies have shown that BCL6 deficiency significantly augments basal as well as inducible pro-inflammatory chemokine and cytokine expression in macrophages [12]. Studies by Barish et al. showed that BCL6 suppresses TLR4-mediated NFκB target gene expression in a nuclear receptor co-repressor 1 (Ncor1)/nuclear receptor co-repressor 2 (Ncor2)-dependent manner [10]. Concordant with these observations, BCL6-deficient mice are highly prone to lethal inflammatory disease conditions due to the overactivation of innate and adaptive immune cell components [34]. Our previous studies have shown that CITED2 limits broad pro-inflammatory gene programs in macrophages [17]. Myeloid-CITED2-deficient mice are highly prone to endotoxin-induced sepsis symptomology [17], HFD-induced atherosclerotic plaque development [18], and zymosan-induced lung inflammation in vivo [19]. Mechanistically, CITED2 curbs pro-inflammatory gene expression by restraining NFkB, HIF1α, STAT1, and IRF1 signaling in macrophages [17,18,19]. In this study, our analyses show that myeloid-CITED2 deficiency promotes HFD-induced adipose tissue inflammation, adverse remodeling of adipocytes, expansion of adipose tissue volume, obesity, insulin resistance, and glucose intolerance. In addition, our analyses also show that HFD feeding substantially attenuated CITED2 as well as BCL6 expression in adipose tissue macrophages that could allow significant expansion of pro-inflammatory gene expression. Previous studies have shown that adipose tissue inflammation is a major mediator of diet-induced obesity and insulin resistance [35]. Thus, attenuation in the CITED2–BCL6 signaling axis in tissue macrophages could result in accelerated development of obesity and metabolic syndrome in hosts fed a high-fat diet. 

Prior clinical and experimental studies have demonstrated that macrophages are the key mediators of chronic inflammation in metabolic organs that contribute to the development of obesity and metabolic syndrome [25]. Specifically, altering the expression or functions of transcription factors in macrophages is known to impact HFD-induced obesity, insulin resistance, and atherosclerosis plaque development [36,37,38]. Particularly, overexpression of BCL6 substantially curtailed HFD-induced metabolic tissue macrophage accumulation, pro-inflammatory gene expression, and lipid deposition [13]. Further, forced expression of BCL6 lowered blood glucose levels as well as HFD-induced insulin resistance [13]. Similarly, myeloid-BCL6 deficiency also robustly elevated HFD-induced vascular inflammatory disease conditions such as atherosclerosis [12]. In this regard, our prior studies have shown that CITED2 serves as a negative regulator of inflammatory gene expression and function in macrophages [17]. Moreover, our prior studies also demonstrated that myeloid-CITED2 deficiency substantially heightened basal as well as diet-induced atherosclerotic plaque development in vivo [18]. In this study, our analyses uncovered that myeloid-CITED2 deficiency resulted in significant amplification of many pro-inflammatory signaling pathways in macrophages. Further, our GSEA and gene expression analysis studies revealed that BCL6 repressive gene targets are substantially enriched and heightened in CITED2-deficient macrophages. Further, our current studies show that CITED2 deficiency significantly attenuated BCL6 mRNA and protein expression in macrophages. This attenuation of BCL6 expression in a CITED2-deficient state allows pro-inflammatory cistromes to augment broad pathogenic gene expression in macrophages.

Previous studies have demonstrated that BCL6 expression is regulated by multiple transcriptional regulators to modulate diverse cellular functions [39]. Particularly, STAT3 has been shown to promote BCL6 expression, and in contrast, STAT5 outcompetes STAT3 to repress BCL6 expression due to shared DNA-binding sites [30]. Our integrated transcriptomics and GSEA studies demonstrated that STAT5-signaling-regulated gene targets are positively enriched in CITED2-deficient macrophages. In this context, previous reports have shown that STAT5 represses BCL6 expression through interaction with CBP/p300 [30]. Further, CBP/p300 has been shown as a coactivator of STAT5 by directly binding to the carboxyl-terminal transactivation domain (TAD) of STAT5 [40]. Interestingly, earlier studies have also shown that the CITED2 transactivation domain (TAD) binds to CBP/p300 with very high affinity [41]. This high-affinity CITED2 binding could displace STAT5 interaction with coactivator CBP/p300, and eventually eject STAT5 from the transcriptional complex, thereby curtailing STAT5 functions to repress BCL6 expression. Moreover, tyrosine-694 phosphorylation of STAT5 is required for dimerization, nuclear transportation, and functional activation of STAT5 [42]. Interestingly, phosphorylation of STAT5 on tyrosine-694 residue is also known to be required for the repressive functions of STAT5 [40]. In this regard, our current findings show that CITED2 deficiency elevates STAT5 phosphorylation (Tyr-694) and attenuates BCL6 expression in macrophages. In addition, our current studies also show that loss of functional STAT5 signaling was sufficient to restore inducible BCL6 expression and attenuate BCL6 repressive target gene expression in CITED2-deficient macrophages. Taken together, these results indicate that CITED2 stifles STAT5 signaling to promote BCL6 expression and BCL6-dependent repression of pro-inflammatory gene expression in macrophages. 

## 5. Conclusions

In summary, our in vitro, ex vivo, and in vivo observations highlighted the importance of the CITED2–BCL6 signaling axis in restraining macrophage inflammatory gene expression and inflammatory disease pathogenesis. Further, this study provided initial evidence that myeloid-CITED2 is protective against HFD-induced obesity, glucose intolerance, and insulin resistance. Mechanistically, CITED2 limits broad pro-inflammatory gene programs by restraining STAT5 signaling to promote BCL6 expression in macrophages. Moreover, genetic inhibition of STAT5 completely reversed elevated pro-inflammatory gene expression in CITED2-deficient macrophages. Thus, our study findings will have implications for numerous chronic inflammatory disease processes, including, but not limited to, inflammatory bowel disease, asthma, arthritis, vasculitis, aneurysm, thrombosis, and peripheral artery diseases.

## Data Availability

The authors confirm that the data supporting the findings of this study are available within the article. Raw data that support the findings of this study are available from the corresponding author upon request.

## Supplementary Materials

The following supporting information can be downloaded at: https://www.mdpi.com/article/10.3390/cells12172136/s1, Figure S1. Interactive gene network of dysregulated signaling pathways in CITED2-deficient macrophages; Figure S2. (A–D) CITED2 deficiency derepresses BCL6 gene targets; Figure S3. GSEA of BCL6 repressive gene targets elevated in CITED2-deficient macrophages; Table S1. Materials and reagents used; Table S2. List of primers used.
