# Supplementary material for: Myeloid-CITED2 Deficiency Exacerbates Diet-Induced Obesity and Pro-Inflammatory Macrophage Response
Source: Cells. 2023 Aug 24;12(17):2136. doi: 10.3390/cells12172136 (PMC10486650; doi:10.3390/cells12172136)
Supplement: Supplementary file 1 [file cells-12-02136-s001.zip › cells-2459253-Supplementary.pdf]

Supplementary Material

Myeloid-CITED2 deficiency exacerbates diet-induced obesity and pro-inflammatory macrophage response.

Atif Zafar, Hang Pong Ng, E. Ricky Chan, Sally L. Dunwoodie, Ganapati H. Mahabeleshwar*

***Correspondence:** Ganapati H. Mahabeleshwar: ghm4@case.edu

Table legends:

Table S1. Materials and reagents used.

Table S2. List of primers used.

Figure legents:

Figure S1. Interactive gene network of dysregulated signaling pathways in CITED2 deficient macrophages. Interactive gene networks between the major inflammatory signaling pathways that are upregulated in CITED2 deficient macrophages were constructed by utilizing Cytoscape 3.9.1. The interactive gene network between HALLMARK inflammatory response, IL2-STAT5-signaling, IFN-γ response, IL6-JAK-STAT3-signaling, hypoxia response,glycolysis pathway, TGF-b signaling, coagulation, TNF-a signaling via NFκB, complement, andIFN-α response are shown. The dysregulated pathways are represented with triangle nodes and dark grey circles showing target genes. The gene interaction across the signaling pathways are shown in the unique color specified to a particular signaling pathway.

Figure S2. (A-D) CITED2 deficiency derepresses BCL6 gene targets. (A-D) Total RNA samples from *Lyz2^{cre}* and *Cited2$^{fl/fl}$:Lyz2^{cre}* mice BMDMs were subjected to RNAseq analysis. The RNAseq data were evaluated for alteration in BCL6 repressive gene targets. The heatmaps of BCL6 repressive gene targets that are significantly upregulated in *Cited2$^{fl/fl}$:Lyz2^{cre}* mice BMDMs as compared to *Lyz2^{cre}* mice BMDMs are shown (n=3).

Figure S3. GSEA of BCL6 repressive gene targets elevated in CITED2 deficient macrophages. The BLC6 repressive gene targets elevated in CITED2 deficient macrophages were subjected to GSEA by utilizing HALLMARK gene sets. The major inflammatory pathways that are significantly enriched are shown. FDR q-value *** $p < 0.001$.

Supplementary Figure S1

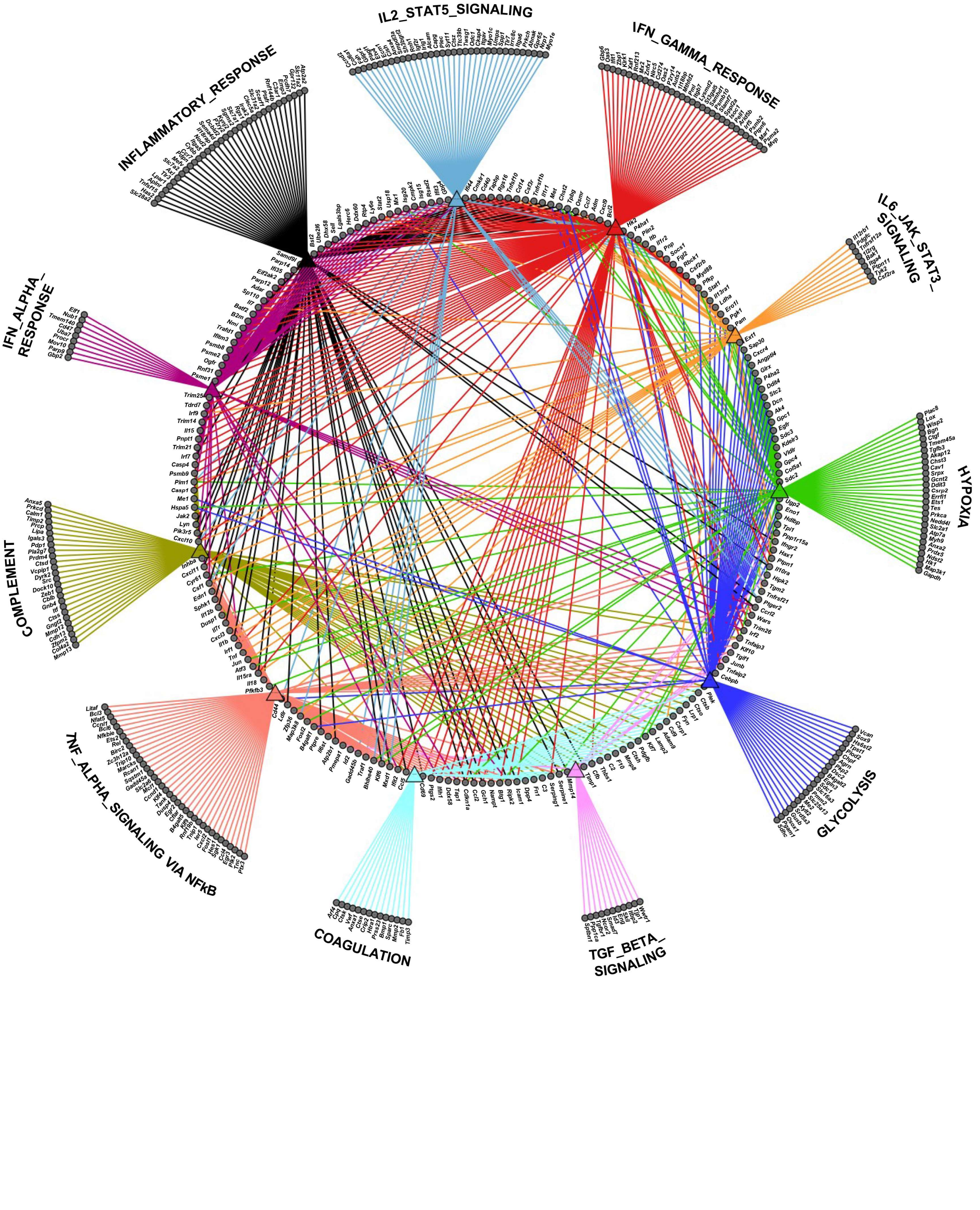

Supplementary Figure S2

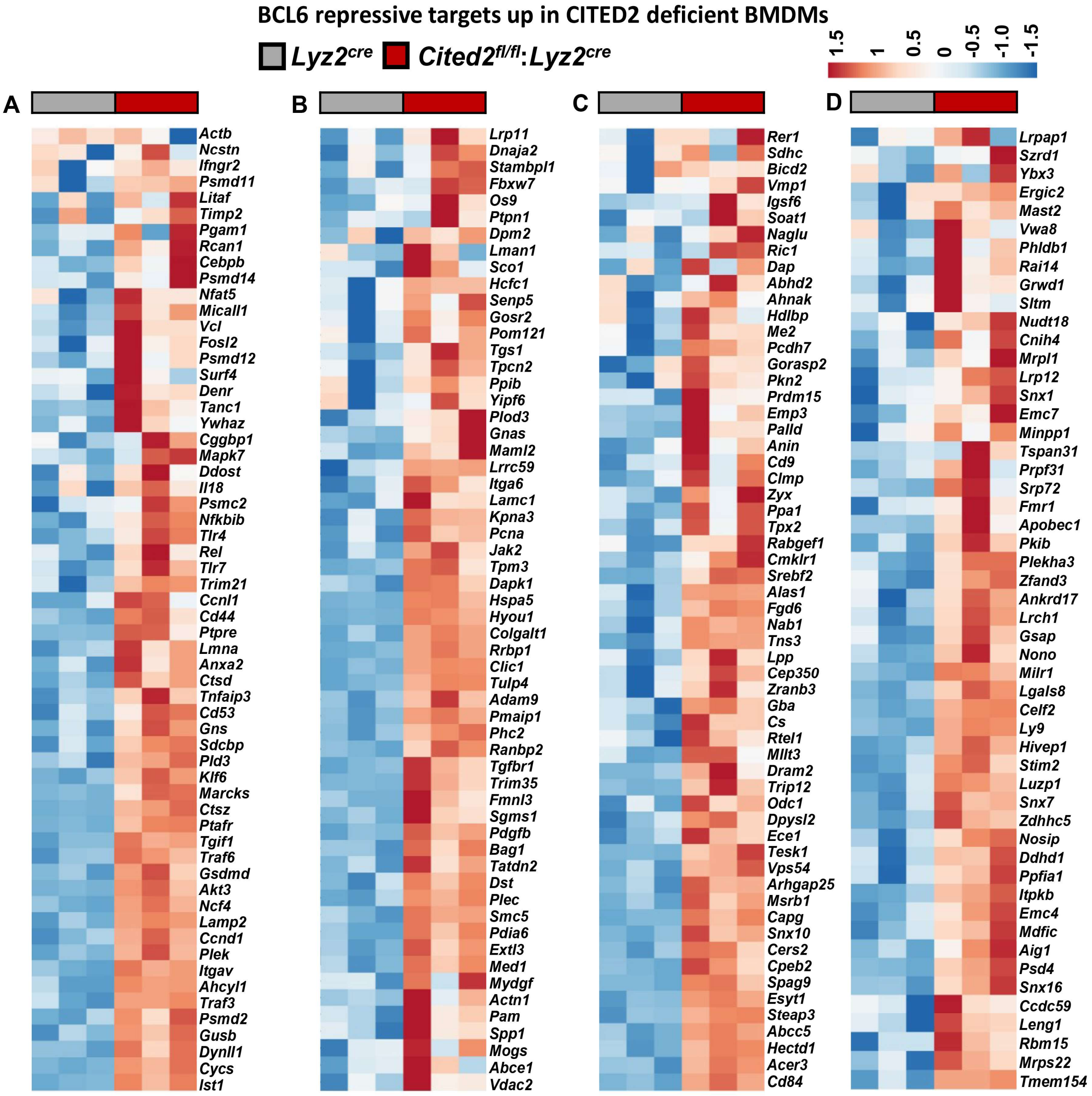

Supplementary Figure S3

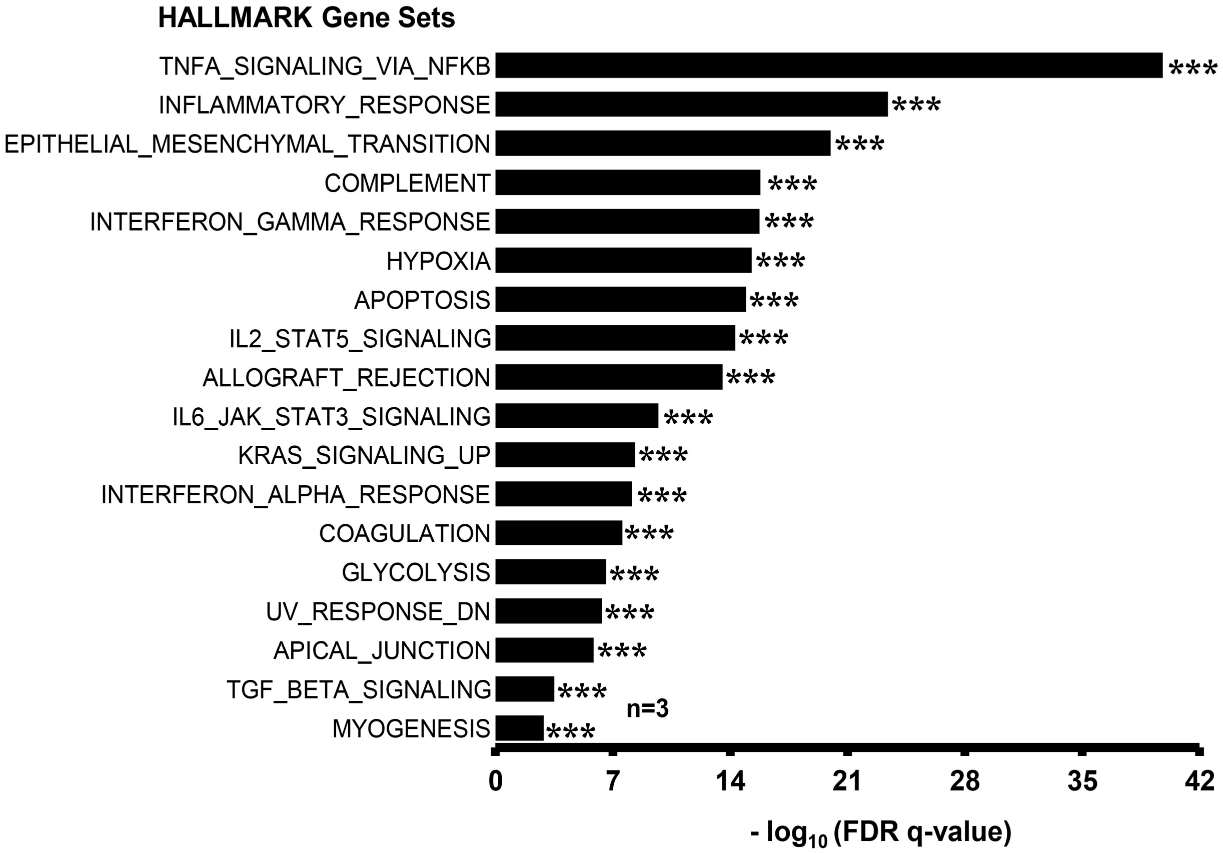

Table S1. Materials and reagents used.

Reagent or resource	Source	Identifier
**Antibodies**		
BCL6	Cell Signaling	Cat # 4242S
Phospho-STAT3 (Tyr705)	Cell Signaling	Cat # 9145S
Phospho-STAT5 (Tyr694)	Cell Signaling	Cat # 4322S
Anti-STAT3	Cell Signaling	Cat # 4904S
Anti-STAT5	Cell Signaling	Cat # 25656S
β-Actin	Santa Cruz	Cat # sc-130656
Rabbit anti-mouse F4/80	Cell Signaling	Cat # 70076S
HRP-conjugated goat anti-rabbit IgG	ThermoFisher Scientific	Cat # A16096
HRP-conjugated goat anti-mouse IgG	ThermoFisher Scientific	Cat # A16066
**Experimental models: Cell Lines**		
RAW264.7	ATCC	Cat # TIB-71
**Experimental models: Organisms/Strains**		
$Lyz2^{cre}$ mice	Jackson Labs	Cat # 004781
$Cited2^{fl/fl}$ *mice*	PMID: 17133411	
$Cited2^{fl/f}:Lyz2^{cre}$ *mice*	PMID: 29203644	
**Recombinant DNA**		
pCMV6-neo-Cited2	Origene	Cat # MC202716
Bcl6-Luc reporter vector		PMID: 17785208
**Sequence-Based Reagents**		
Random hexamers	ThermoFisher Scientific	Cat # N8080127
Oligo-dT primer	ThermoFisher Scientific	Cat # 18418020
siSTAT5 (FlexiTube siRNA)	Qiagen	Cat # GS6776
siCited2 siRNA	GE-Dharmacon	Cat # L-044387-01
**Other reagents**		
High glucose DMEM	GE-Healthcare Life Sciences	Cat # SH30249.02
1x PBS	GE-Healthcare Life Sciences	Cat # SH30028.03
Penicillin, Streptomycin, glutamic acid	ThermoFisher Scientific	Cat # 10378-016
FBS	Atlanta Biologicals	Cat # S11550
Recombinant Mouse M-CSF	R&D Systems	Cat # 416-ML
Lipopolysaccharide	InvivoGen	Cat # tlrl-pb5lps
High Pure RNA Isolation Kit	Roche	Cat # 11828665001
M-MuLV reverse transcriptase	New England Biolabs	Cat # M0253L
Fast SYBR Green PCR Master Mix	ThermoFisher Scientific	Cat # 4385612

TaqMan Fast Advanced Master Mix	ThermoFisher Scientific	Cat # 4444556
Hematoxylin	ThermoFisher Scientific	Cat # 7211
Eosin	ThermoFisher Scientific	Cat # 71304
10% neutral buffered formalin	MilliporeSigma	Cat # HT5012-1CS
Antigen unmasking solution	Vector Labs	Cat # H-3300
ABC reagent	Vector Labs	Cat # PK-4000
DAB reagent	Vector Labs	Cat # SK-4100
anti-F4/80 microbeads	Miltenyi Biotec	Cat# 130-110-443
Lipofectamine3000	Thermo Fischer Scientific	Cat # L3000-008
Dual-Luciferase reporter assay	Promega Corp	Cat # E1960
RIPA buffer	Sigma-Aldrich	Cat # R0278
Protease inhibitor	Roche	Cat # 04693132001
Phosphatase inhibitor	Roche	Cat # 04906837001
BCA assay	Thermo Fisher Scientific	Cat # 23227
Nitrocellulose membranes	GE-Healthcare Life Sciences	Cat # 10401197
Non-fat dry milk	Bio-Rad	Cat # 1706404XTU
Bovine serum albumin	Sigma-Aldrich	Cat # A3608
TBS-T	Boston BioProducts	Cat #IBB-181
ECL Western Blotting Substrate	Thermo Fisher Scientific	Cat # 32106
X-ray films	Denville Scientific	Cat # E3018

Table S2. List of primers used.

Target Gene	Forward Primer	Reverse Primer
Cited2	5'-TTGGACCGCATCAAGGAG-3'	5'-GCACACGAAGTCCGTCATA-3'
Bcl6	5'-TGCAGATGGAGCATGTTGTC-3'	5'-TTCACGGGGAGGTTTAAGTG-3'
Ccl7	5'-GCTGCTTTCAGCATCCAAGTG-3'	5'-CCAGGGACACCGACTACTG-3'
Cxcl9	5'-TCCTTTTGGGCATCATCTTCC-3'	5'-TTTGTAGTGGATCGTGCCTCG-3'
Usp18	5'-GCTTGACTCCGTGCTTGAG-3'	5'-CGGGAGTCCACAACTTCACT-3'
C3	5'-CCAGCTCCCCATTAGCTCTG-3'	5'-GCACTTGCCTCTTTAGGAAGTC-3'
Cish	5'-ATGGTCCTTTGCGTACAGGG-3'	5'-GGAATGCCCCAGTGGGTAAG-3'
Ccrl2	5'-GCCCCGGACGATGAATATGAT-3'	5'-CACCAAGATAAACACCGCCAG-3'
F10	5'-CTGCTCGGAGGGATTTGA-3'	5'-TGTCTAGGCGGCAGAGTTTC-3'
Inhba	5'-TGAGAGGATTTCTGTTGGCAAG-3'	5'-TGACATCGGGTCTCTTCTTCA-3'
Ccnd2	5'-GAGTGGGAACTGGTAGTGTTG-3'	5'-CGCACAGAGCGATGAAGGT-3'
Csf1	5'-ATGAGCAGGAGTATTGCCAAGG-3'	5'-TCCATTCCCAATCATGTGGCTA-3'
Irf7	5'-GAGACTGGCTATTGGGGGAG-3'	5'-GACCGAAATGCTTCCAGGG-3'
Serpine1	5'-AGGATCGAGGTAAACGAGAGC-3'	5'-GCGGGCTGAGATGACAAA-3'
Oasl1	5'-GAGACAGCTCAGGGGACAAA-3'	5'-GAGCCACTATGTCCCATCTGTA-3'
Mx1	5'-TTCAAGGATCACTCATCATTCAGC-3'	5'-GGGAGGTGAGCTCCTCAGT-3'
Nampt	5'-GCAGAAGCCGAGTTCAACATC-3'	5'-TTTTCACGGCATTCAAAGTAGGA-3'
Prdm1	5'-GAGGATCTGACCCGAATCAA-3'	5'-GTTGCTGTGAGGCAACTTCA-3'
Cd40	5'-TGTCATCTGTGAAAAGGTGGTC-3'	5'-ACTGGAGCAGCGGTGTTATG-3'
Il1a	5'-GCACCTTACACCTACCAGAGT-3'	5'-AAACTTCTGCCTGACGAGCTT-3'
Tnf	5'-CCCTCACACTCAGATCATCTTCT-3'	5'-GCTACGACGTGGGCTACAG-3'
Ccl2	5'-TTAAAAACCTGGATCGGAACCAA-3'	5'-GCATTAGCTTCAGATTTACGGGT-3'
Il12b	5'-TGGTTTGCCATCGTTTTGCTG-3'	5'-ACAGGTGAGGTTCACTGTTTCT-3'
Mrc1	5'-GGACGAGCAGGTGCAGTT-3'	5'-CAACACATCCCGCCTTTC-3'
Arg1	5'-TTTTAGGGTTACGGCCGGTG-3'	5'-CCTCGAGGCTGTCCTTTTGA-3'
Chil3l3	5'-CAGGTCTGGCAATTCTTCTGAA-3'	5'-GTCTTGCTCATGTGTGTAAGTGA-3'
Retnla	5'-CCCTCCACTGTAACGAAGACTC-3'	5'-CACACCCAGTAGCAGTCATCC-3'
36B4	5'-GCTCCAAGCAGATGCAGCA-3'	5'-CCGGATGTGAGGCAGCAG-3'
